# A Note on “Malaria at Parturition in Nigeria: Current Status and Delivery Outcome”

**DOI:** 10.1155/2009/426201

**Published:** 2010-02-14

**Authors:** Kazeem A. Oshikoya, Idowu O. Senbanjo

**Affiliations:** ^1^Department of Pharmacology, Lagos State University College of Medicine, P.M.B 21266, Ikeja, Lagos, Nigeria; ^2^Department of Paediatrics, Lagos State University Teaching Hospital, Ikeja, Lagos, Nigeria; ^3^Academic Division of Child Health, University of Nottingham, The Medical School, Royal Derby Children's Hospital, Uttoxeter Road, Derby DE22 3DT, UK

## Abstract

We read the recent article by (Mokuolu et al. 2009) with keen interest and would like to congratulate them for a job well done. However, we would like to raise a few points relating to the limitations of the study.

Antimalarial use in pregnancy is an important determinant of the level of malaria parasitaemia at delivery but Mokuolu et al. [1], like the other workers from Nigeria and other African countries [2–4], did not assess the effects of antimalarial chemoprophylaxis used during pregnancy on the levels of malaria parasitaemia at parturition. A national guideline for preventing malaria in pregnancy may not have existed in Nigeria in 2003 and 2004 when Mokuolu et al. performed their study; the guidelines of the World Health Organization (WHO) was in existence and was well known to many of the obstetricians in Nigeria [4]. Onah et al. [4] reported that 86% of the obstetricians surveyed at the annual conference of the Society of Obstetrics and Gynaecology of Nigeria (SOGON) in 2001 offered chemoprophylaxis to their patients. The chemoprophylaxis includes chloroquine, pyrimethamine, proguanil, and sulphadoxine-pyrimethamine. Similarly, a study conducted by Falade et al. [5] in 2003 and 2004, about the same time Mokuolu et al. did their study, showed that 85.5% of pregnant women used some form of antimalarial prophylaxis, sulphadoxine-pyrimethamine being the most frequently used drug. Self-medication is very rampant in Nigeria and antimalarials, especially sulphadoxine-pyrimethamine, being one of the drugs most frequently self-medicated [6]. Pregnant women may practice self-medication of antimalarials, moreso when antimalarials are categorised as over-the-counter drugs. Therefore, it is very likely that Mokuolu's patients had used some form of antimalarials prior to their evaluation and may not have volunteered such history if their antimalarial drug history was focused only on chemoprophylaxis. 

The authors claimed that they took specific note of those patients who used preventive antimalarial drugs, yet they did not provide the proportion of such patients. These patients are likely to procure the drug from the community pharmacies and are at risk of using counterfeit and adulterated antimalarials which are very abundant in Nigeria [7]. In the study of Tako et al. [8], 76.3% of their patients had used one form of antimalarial chemoprophylaxis, yet 21.4% of the patients had malaria parasitaemia at delivery. The failure rate was attributed to resistance to the drugs or to lack of efficacy. Considering the risk of resistance and threats to efficacy of antimalarials used for chemoprophylaxis in pregnancy in Nigeria, it would have been proper for the authors to ascertain the use of antimalarials by their patients through assay of blood levels of the drugs. This may be a better way to eliminate the influence of antimalarial drugs on the level of parasitaemia observed in their study rather than relying on the medication history of the patients. 

The inclusion criteria used in the study appear not to be clearly defined by the authors. We are not sure if pregnant women with HIV infection and unbooked patients were part of the cohorts they studied. Perhaps, it would have been proper to exclude these groups of patients because of the possible bias their inclusion may introduce into the study and this may be better avoided by recruiting patients from antenatal clinics and followed to the time of delivery rather than recruiting at the point of delivery. HIV and malaria coinfection has more adverse effects on pregnancy than malaria alone [9]. The prevalence and intensity of malaria infection during pregnancy are higher among HIV-infected women and so is the risk to the woman and her newborn, irrespective of her gravidity [10]. Coinfection more than doubled the risk of moderate to severe anaemia in all pregnant women [11], meaning that a considerable proportion of children born to mothers with both HIV and malaria are more likely to be at risk of low birth weight and infantile mortality. Unbooked patients are likely to receive antenatal care from traditional birth attendants, primary health care centres, or private practitioners who are not knowledgeable about prevention and control of malaria in pregnancy [12, 13]. Therefore, malaria parasitaemia is likely to be more prevalent at delivery in unbooked than booked patients. 

Mokuolu et al. found a significant association between malaria parasitaemia at delivery and maternal age less than twenty years. It may be true that pregnancy associated with acquired immunity is lower in younger women than older ones who have obtained adequate immunity from repeated exposures to malaria infections. Teenage pregnancies are likely to be associated with poverty, lack of good education, and poor nutrition. The necessary proteins needed in the synthesis of antibodies against malaria parasites may therefore be reduced in young pregnant women and may indirectly contribute to the high prevalence of malaria parasitaemia observed by the authors in young parturients.

Given that the authors are from various disciplines, a future study that will involve a multidisciplinary approach is therefore suggested. The ability of the contributing authors, who are clinical pharmacologists, to assay blood levels of antimalarial drugs in pregnancy will add novelty to the future study.

## References

[1] Mokuolu OA, Falade CO, Orogade AA (2009). Malaria at parturition in nigeria: current status and delivery outcome. *Infectious Diseases in Obstetrics and Gynecology*.

[2] Ibhanesebhor SE, Okolo AA (1992). Placental malaria and pregnancy outcome. *International Journal of Gynecology and Obstetrics*.

[3] Sule-Odu AO, Ogunledun A, Olatunji AO (2002). Impact of asymptomatic maternal malaria parasitaemia at parturition on perinatal outcome. *Journal of Obstetrics and Gynaecology*.

[4] Onah HE, Nkwo PO, Nwakwo TO (2006). Malaria chemoprophylaxis during pregnancy: a survey of current practice amongst Nigerian obstetricians. *Tropical Journal of Obstetrics and Gynaecology*.

[5] Falade CO, Yusuf BO, Fadero FF, Mokuolu OA, Hamer DH, Salako LA (2007). Intermittent preventive treatment with sulphadoxine-pyrimethamine is effective in preventing maternal and placental malaria in Ibadan, south-western Nigeria. *Malaria Journal*.

[6] Oshikoya KA, Njokanma OF, Bello JA, Ayorinde EO (2007). Family self-medication for children in an urban area of Nigeria. *Paediatric and Perinatal Drug Therapy*.

[7] Ibekwe J (2009). Artemesinin resistant malaria on the horizon. *International Journal of Medicine and Medical Sciences*.

[8] Tako EA, Zhou A, Lohoue J, Leke R, Taylor DW, Leke RFG (2005). Risk factors for placental malaria and its effect on pregnancy outcome in Yaounde, Cameroon. *American Journal of Tropical Medicine and Hygiene*.

[9] Briand V, Badaut C, Cot M (2009). Placental malaria, maternal HIV infection and infant morbidity. *Annals of Tropical Paediatrics: International Child Health*.

[10] Verhoeff FH, Brabin BJ, Hart CA, Chimsuku L, Kazembe P, Broadhead RL (1999). Increased prevalence of malaria in HIV-infected pregnant women and its implications for malaria control. *Tropical Medicine and International Health*.

[11] Ayisi JG, van Eijk AM, ter Kuile FO (2003). The effect of dual infection with HIV and malaria on pregnancy outcome in western Kenya. *AIDS*.

[12] Omo-Aghoja LO, Abe E, Feyi-Waboso P, Okonofua FE (2008). The challenges of diagnosis and treatment of malaria in pregnancy in low resource settings. *Acta Obstetricia et Gynecologica Scandinavica*.

[13] Ofili AN, Okojie OH (2005). Assessment of the role of traditional birth attendants in maternal health care in Oredo Local Government Area, Edo State, Nigeria. *Journal of Community Medicine and Primary Health Care*.

